# Quantitative assessment of retinal thickness and vessel density using optical coherence tomography angiography in patients with Alzheimer’s disease and glaucoma

**DOI:** 10.1371/journal.pone.0248284

**Published:** 2021-03-19

**Authors:** Przemyslaw Zabel, Jakub J. Kaluzny, Katarzyna Zabel, Martyna Kaluzna, Aleksander Lamkowski, Damian Jaworski, Jaroslaw Makowski, Martyna Gebska-Toloczko, Robert Kucharski

**Affiliations:** 1 Department of Sensory Organ Studies, Nicolaus Copernicus University, Collegium Medicum, Bydgoszcz, Poland; 2 Oftalmika Eye Hospital, Bydgoszcz, Poland; 3 Department of Ophthalmology, Collegium Medicum, Nicolaus Copernicus University, Bydgoszcz, Poland; 4 Center of Psychoneurology in Elderly, Sue Ryder Home leading by Pallmed Ltd, Bydgoszcz, Poland; University of Florida, UNITED STATES

## Abstract

**Purpose:**

Assessment and a direct comparison of retinal vessel density with the thickness of inner retinal layer (IRL) and outer retinal layer (ORL) in the same regions of the macula in subjects with Alzheimer’s disease (AD) and primary open-angle glaucoma (POAG).

**Methods:**

We analyzed data from 48 eyes of healthy control (HC) participants, 71 eyes with POAG, and 49 eyes of AD patients. Ophthalmic examination included optical coherence tomography (OCT) imaging to measure IRL and ORL thickness and OCT angiography (OCTA) in the same region for the imaging of vessel density in the superficial vascular plexus (SVP) and deep vascular plexus (DVP) of the retina. A direct comparison of vessel density and retinal layers thickness, which different dynamic ranges, was obtained by normalizing values as percentage losses.

**Results:**

Patients with AD presented significantly greater losses of vascular density in the DVP and ORL thickness compared to POAG (p <0.001), but percentage losses of vessel density in SVP and IRL thickness were considerable in POAG compared to AD eyes (p<0.001). Positive associations among presence of AD were observed primarily in outer retina where a 1% decrease of ORL thickness was associated with about 24–29% increase in odds of the presence of AD. According to OCTA measurements, a 1% decrease of vessel density in DVP was positively associated with a 4–9% increase in odds of the presence of AD. In POAG positive associations among presence of disease were observed only in inner retina where 1% loss of IRL thickness and a 1% loss of vessel density in the SVP were positively associated with a 13–23% increase in risk of presence of the disease.

**Conclusions:**

Analysis of ORL thickness and vessel density in DVP could potentially improve diagnostic capabilities and may provide a valuable approach for predicting of AD.

## Introduction

The leading cause of dementia is Alzheimer’s disease (AD), characterized by chronic inflammation, glial disorders, and synaptic loss in the central nervous system which begin decades before the disease is fully clinically expressed [1]. In 2018, the National Institute on Aging and the Alzheimer’s Association (NIA-AA) proposed a new research framework for AD as a means of diagnosing and staging AD in living people. Biomarkers are grouped into those related to amyloid-β (Aβ) deposition, pathological aggregation of phosphorylated Tau (pTau), and neurodegeneration (ATN). This classification system groups and evaluates various biomarkers using neuroimaging, e.g., structural magnetic resonance imaging (MRI), positron emission tomography (PET), or molecular measurement of protein levels in cerebrospinal fluid (CSF) [2]. However, existing modalities for diagnosing AD present several disadvantages, such as a lack of standardization and invasiveness in the case of CSF markers and high costs and currently limited availability of PET imaging. In addition, there are still doubts as to whether current methods are sensitive and specific enough to establish a definitive diagnosis of AD [3,4].

During embryogenesis, the retina develops as a direct extension of the diencephalon and cranial nerve (CN) II. Retinal ganglion cell (RGC) axons do not have specific features of peripheral nerves and are essentially white matter surrounded by meninges [5]. The first histological studies more than 30 years ago reported changes in the CNII and retina as a result of neurodegenerative changes in the brain of AD patients [6]. Anatomical alterations such as loss of RGC leading to a reduction in thickness of ganglion cell layer (GCL) and retinal nerve fiber layer (RNFL) in eyes of patients with AD were confirmed in subsequent reports [7,8]. However, RGC apoptosis occurs not only in AD but in other neurodegenerative diseases, particularly in glaucoma [9,10].

Since the 1990s when optical coherence tomography (OCT) was introduced, measurements of RNFL and GCL thickness have become parameters commonly used in the diagnosis and monitoring of glaucoma [11]. It is notable, that recent studies using OCT found that values of RNFL, and also GCL thickness were reduced in patients with AD when compared with healthy subjects, but that thickness was even more decreased in eyes with primary open-angle glaucoma (POAG) [12–15]. Therefore, it seems that the thickness measurements of the RNFL and the inner retinal layers (IRL) of the macula, which have been previously proposed as surrogate markers for the identification and monitoring of AD, are not specific enough to be used in everyday practice.

Post-mortem histopathological brain examinations of patients with AD have shown that the disease also causes cerebrovascular pathology, however, changes in the microvasculature of the CNS remain difficult to investigate *in vivo* [16]. Blood vessels of the retina and brain share a common embryological origin and display similar anatomical and physiological properties, thus retinal vascular examination may provide new, valuable information on AD [17]. With the introduction of OCT angiography (OCTA), a modern technique for non-invasive imaging of retinal blood vessels *in vivo*, it has been demonstrated that retinal vessel density is significantly reduced in patients with AD, likely due to abnormal Aβ deposits that build up inside blood vessel walls [18–21]. Latest studies demonstrated significant correlations between retinal vessel density and cognitive function domains [22,23], apolipoprotein E genotype AD [24], and cerebral volumetric changes [25]. However, OCTA imaging has also provided evidence of microvascular impairment owing to reduced vessel density within the peripapillary area and the macula in POAG [26].

Although new imaging technologies, such as OCT and OCTA, have advanced our understanding of the pathophysiology of AD, identifying which biomarkers of the eye are most useful in the diagnosis of AD remains challenging, moreover, it is difficult to distinguish AD from other neurodegenerative diseases, primarily POAG, with sufficient accuracy.

The purpose of the present study was to characterize and perform a direct comparison of retinal vessel density with the thickness of IRL and outer retinal layer (ORL) in the same regions of the macula in subjects with AD and POAG. In addition, we used spectral-domain OCT (SD-OCT) and OCTA to determine the associations of changes in vessel density and retinal layers thickness with the presence of AD and POAG.

## Materials and methods

### Study design and subjects

This was a cross-sectional study carried out between January 2018 and March 2019 in the Oftalmika Eye Hospital in Bydgoszcz, Poland. The research was conducted in accordance with the principles of the Helsinki Declaration. The protocol of the study was approved by the Bioethical Commission of Nicolaus Copernicus University in Torun, Collegium Medicum in Bydgoszcz (approval number: 473/2017). Written informed consent was obtained from all participants. Each participant enrolled in the study was examined by a psychologist and cognitive function was assessed using the Mini-Mental State Examination (MMSE) screening test. An interview with each subject was conducted by a physician to obtain demographic information, medical and neurologic history, and a risk factor profile. Patients with AD remained under the care of their psychiatrist, who determined that the participants are able to express informed consent. All patients included in the study underwent a detailed ophthalmological examination which included: best-corrected visual acuity (BCVA) assessment, tonometry (Icare TAO1i, USA), slit-lamp biomicroscopy to assess iridocorneal angle, and dilated fundus examination. Thickness of the peripapillary RNFL (pRNFL) and retinal macular region were measured using SD-OCT. Retinal vessel density was assessed in the same region using OCTA. Examinations were carried out over one day by a single ophthalmologist.

The group of AD patients were referred from the Psychoneurology of the Elderly Center in Bydgoszcz. Each of them remained under the care of this center for at least a year, where, in addition to cognitive therapy, they received drugs. In the mild stage of the disease, these were acetylcholinesterase inhibitors, while in the moderate stage, they were NMDA receptor antagonists or combination therapy. AD was diagnosed by a psychiatrist physician according to the Diagnostic and Statistical Manual of Mental Disorders (DSM-IV) and the NIA–AA criteria [27]. To confirm the presence of fibrillar brain amyloid, PET imaging with florbetapir F 18 radioligand was performed. Images were constructed using the standard uptake value ratio (SUVr) based on 90–110 min of acquired data. Global values were computed based on the volume weighted average of frontal (superior, middle, and inferior frontal gyrus), parietal (posterior cingulate, superior parietal gyrus, postcentral gyrus, and inferolateral remainder of parietal lobe), and temporal (parahippocampal gyrus, hippocampus, medial temporal lobe, superior, middle, and inferior temporal gyrus) regions. All SUVr images were visually read by an experienced nuclear physician. If SUVr was > 1.5, AD subjects were classified as amyloid-positive [28]. Patients with mild to moderate dementia (MMSE score, 10–23) qualified for entry into the study. Additional inclusion criteria were a normal intraocular pressure (IOP; < 21 mmHg) and the absence of ocular fundus changes suggestive of glaucoma. Due to poor cooperation affects to the low reliability of static perimetry test in patients with AD, the examination was not performed in this group of patients.

Patients with POAG and cognitively normal according to neuropsychological assessment were enrolled in the study based on the presence of features of glaucoma optic neuropathy, accompanied by a decrease in pRNFL thickness corresponding to loss of visual field based on standard automated perimetry (SITA Standard 24–2, Humphrey Field Analyser II, Carl Zeiss Meditec). Glaucomatous visual filed damage was defined as a glaucoma hemifield test (GHT) outside normal limits and a pattern standard deviation (PSD) outside the 95% normal limit, confirmed by at least two consecutive reliable tests (fixation losses ≤ 33%, false-positives, and false-negatives ≤ 20%). Patients in stage 1 or 2 glaucomatous damage were included in the study [29]. Each eye (100%) in the POAG group was treated with at least one type of ocular antihypertensive drops. The mean number of antihypertensive eye drops was 1.6 ± 0.7. The most commonly used were beta-blockers (59.2%), followed by prostaglandin analogues in 36.6%, carbonic anhydrase inhibitors in 33.8%, and alpha2-adrenergic receptor agonists in 30.9%.

Participants in the healthy control group had an IOP of less than 21 mmHg, normal optical nerve head (ONH) images without asymmetry, pRNFL thickness within normal limits, normal results in visual field examination, defined as a PSD within the 95% confidence interval (CI), and a GHT result within normal limits. Control subjects were ascertained to be cognitively normal according to neuropsychological assessment.

General exclusion criteria included: age below 50 and above 85, BCVA ≤ 0.6, refractive defect above ± 3.0 Dsph, IOP > 23 mmHg, ocular trauma, vascular or non-vascular retinopathies, non-glaucomatous optic neuropathies, macular disease with a history of eye surgery, except uncomplicated cataract phacoemulsification for all groups and uncomplicated anti-glaucoma surgery only for the POAG group when at least 3 months have passed since surgery. People with neurodegenerative diseases other than AD, a history of alcohol abuse or carbon monoxide poisoning, or other serious chronic medical conditions affect the vascular system, such as diabetes, thyroid disease, uncontrolled arterial hypertension were also excluded from the study.

### OCTA and SD-OCT acquisitions

The study used the RTVue XR Avanti (Optovue Inc., Fremont, CA, USA) SD-OCT device with AngioVue software (version 2017.1.0.151), which provides non-invasive visualization of the retinal vascular network using the split-spectrum amplitude-decorrelation angiography (SSADA) algorithm. The system is implemented on an existing commercially available SD-OCT platform that provides both retinal thickness and vessel density measurements. By simultaneously acquiring the OCT and OCTA volume of the AngioVue scan and using automatic segmentation, both the vessel density and thickness can be obtained from the same scan with accurate registration of analyzed areas. The OCTA device has the ability to perform 70,000 A-scans per second and allows measurements with an axial resolution of 5 μm using a light source with a wavelength of 840 ± 10 nm and a bandwidth of 45 nm. To correct motion artifacts, OCTA combines orthogonal fast-scan directions (horizontal and vertical) and is equipped with DualTrac Motion Correction Technology [30]. The software is equipped with a three-dimensional Projection Artifact Removal algorithm to reduce projection artifacts in deeper layers from the OCTA volume on a per voxel basis using information from the OCT and OCTA volumes to differentiate the OCTA signal from projection artifacts *in situ* [31].

The protocol for macular scanning consisted of B-scans covering a 6x6-mm area repeated horizontally and vertically. Each B-scan contained 400 A-scans with the center located at the fixation point. Scanned images of the 4.5x4.5-mm areas centered on the ONH also consisted of two sets of B-scans repeated horizontally and vertically, each consisting of 400 A-scans. For further analysis, only good technical measurements with a scan quality (SQ) index of 6 or higher on a 10-point scale with which a commercial device was equipped, qualified. Measurements with motion artifacts on en face images (irregular patterns of vessels or a blurred boundary of the ONH) were discarded, as well as those with poor segmentation of individual vascular plexuses.

### Vessel density analysis

The study was conducted on all patients between 10:00 and 16:00 following pupil dilation. Data analyses, including automatic segmentation of the superficial vascular plexus (SVP) and deep vascular plexus (DVP) of the macula and the peripapillary radial peripapillary capillary (pRPC) layer in the ONH area, followed by automatic measurements of vessel density, were performed on commercially available software. Vessel density was calculated as the percentage of area occupied by flowing blood vessels in the selected region. For ONH scans, vessel density was analyzed in the peripapillary area, which extends outwards from the ONH border with an elliptical area between 2–4 mm. The RPC layer was defined as extending from the inner limiting membrane (ILM) to the posterior border of the RNFL. In the macula, analyses of vessel density and retinal thickness were performed on the entire surface of 6x6-mm en face images, inner circle of the Early Treatment of Diabetic Retinopathy Study chart (i.e., foveal area, 1-mm diameter circle), parafoveal area (rings between 1 mm and 3 mm from the center of the fovea), perifoveal area (rings between 3 mm and 6 mm from the center of the fovea), and its sectors. The SVP comprised the area between the ILM and the outer boundary of the inner plexiform layer (IPL), while the DVP comprised the area between the outer boundary of the IPL and the outer boundary of the outer plexiform layer (OPL).

### Thickness analysis of retinal layers

**Fig 1:**
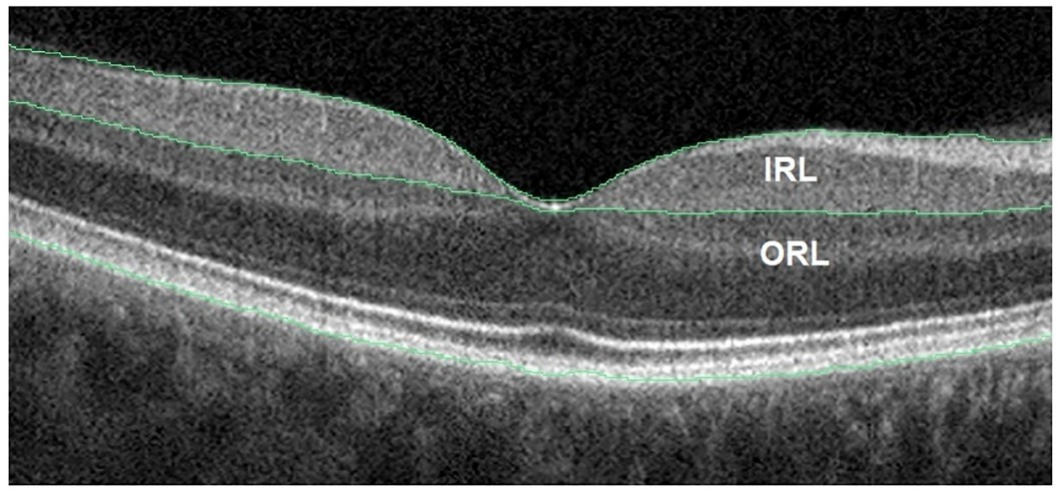
Representative optical coherence tomography image of an eye in a patient with Alzheimer’s disease. Cross-sectional image along the horizontal meridian showing the segmentation boundaries of the inner retinal layer (IRL) and outer retinal layer (ORL) (green lines).

Thickness of the retinal layers was evaluated using the same 6x6-mm and 4.5x4.5-mm OCTA acquisitions used for the vessel density analysis. Mean retinal thickness in the peripapillary and foveal, parafoveal, and perifoveal areas were output by the software. Furthermore, the software automatically segmented the pRNFL as well as the IRL and ORL of the macula. The IRL includes the RNFL, GCL, and IPL, whereas the ORL includes layers starting from the inner nuclear layer (INL) up to the outer portion of the hyper-reflective line corresponding to the retinal pigment epithelium (RPE) (Fig 1).

### Statistical analysis

Summary statistics for normally distributed continuous variables are presented as mean ± one standard deviation (SD) and median with interquartile range (IQR) for non-normally distributed variables. Categorical variables are presented as frequencies. Differences between continuous, normally distributed variables were analyzed using Student’s t-test or analysis of variance (ANOVA) with Bonferroni adjustment for multiple tests. Differences among non-normally distributed data were assessed using the Wilcoxon or Kruskal-Wallis test. When multiple patient groups were compared, multiple testing corrections were applied. Differences between categorical variables were assessed using the chi-square test or Fisher’s exact test for independence. To compare distributions of vessel density and retinal layer thickness in HC, AD, and POAG groups, the linear mixed effect model (LMM) was used, which takes into account the correlation between repeated observations for the same individual (inter-eye correlation).

A direct comparison of vessels density and retinal layers thickness, which different dynamic ranges, was obtained by normalizing values as percentage losses. For patients from the AD and POAG groups, the percentage loss of retinal structure compared to the HC was obtained for the considered variables, i.e., vessel density in the SVP and DVP and thickness of the IRL and ORL calculated using the LMM model [32]. Values of percentage losses were adjusted for inter-eye correlation, age, gender, and SQ, where applicable.

The LMM model was applied to study the association between percentage losses of vessel density and thickness of retinal layers, with thickness as the dependent variable and vessel density, age, gender, and SQ as independent variables. Results are reported as the coefficient of determination (R2), which evaluates the amount of variance in the dependent variable explained by the model. To investigate associations between the percentage losses of considered variables and the presence of AD and POAG, generalized estimating equations (GEE) for correlated multinomial responses were applied [33]. Results are expressed as odds ratios with 95% CI per 1% loss of function.

Results are considered statistically significant when the p-value is less than 0.05. Statistical analyses were performed in the R software (version 3.6.2) using the gls function in the lme4 package, r2glmm package, and multgee package.

## Results

This study initially enrolled 112 subjects (179 eyes) who met the inclusion criteria. Due to poor image quality (motion artifacts, vitreous floaters, incorrect segmentation) in OCTA and SD-OCT examinations, two eyes from the HC group, four eyes from the POAG group, and five eyes from the AD group were excluded. Analyses were carried out on data from 31 HC participants (48 eyes), 46 POAG patients (71 eyes), and 31 AD patients (49 eyes). When both eyes of the same patient were included in the study, we controlled for correlation between same-patient eyes.

Demographic and clinical characteristics of study subjects are summarized in Table 1. There were no significant differences among groups in terms of age, gender, BCVA, and SQ index (p>0.05). The IOP of eyes differed between groups (p = 0.013), with the highest scores reported for POAG. In all eyes with POAG, the disease was of a perimetric nature (mean deviation (MD) -4.7 (-8.2–2.4) dB), whereas eyes in the HC had normal results on visual field in standard automated perimetry, (MD -1.1 (-2.2–0.2) dB) (p<0.001). The MMSE score was significantly lower in AD patients (p<0.001) and median (interquartile range) was 20.5 (18.5–24.5). In the groups of POAG and HC patients, the MMSE score was within the normal range. Thickness of the pRNFL were significantly different among the three groups (p<0.001) with the thinnest mean pRNFL measurement in the POAG group and thickest mean measurement in the HC group. Despite decreased pRNFL thickness (p = 0.038), patients with AD did not exhibit any differences in vessel density in the pRPC (p = 0.906) compared to the HC group, whereas pRNFL thickness was significantly reduced in the POAG group (p<0.001 for POAG vs. AD and POAG vs. HC).

**Table 1:** Demographics and clinical characteristics of participants.

Parameter	Healthy	POAG	AD	P-Value†
**Number of eyes (patients)**	48(31)	71(46)	49(31)	
**Age (years)**	71.4±9.1	72.1±8	74.4±6.1	0.287
**Gender (Male/Female)**	9/23	23/23	9/22	0.747
**MMSE (points)**	29(28–29)*	29(28–30)*	20.5(18.5–24.5)*	**<0.001**
**BCVA (Snellen)**	1(1–1)*	1(1–1)*	1(1–1)*	0.082
**IOP (mmHg)**	18±2.5	18.2±2.3	16.9±1.8	**0.013**
**pRPC (%)**	51±2.8	41.6±7.3	50.7±3.8	**<0.001**
**pRNFL (μm)**	109.2±10.5	78.8±14.5	102.9±13.8	**<0.001**
**SQ index Macula**	8(7–8)*	7(7–8)*	7(7–8)*	0.063
**SQ index Optic Disc**	8(8–9)*	8(7–9)*	8(7–9)*	0.062

Significant values appear in boldface.

Mean (standard deviation).

*Median (interquartile range).

^†^Statistical significance tested by ANOVA or Kruskal-Wallis test (for continuous variables) and by chi-square or Fisher exact test (for categorical variables).

Abbreviations: POAG = primary open-angle glaucoma, AD = Alzheimer’s disease, MMSE = Mini-Mental State Examination, BCVA = best corrected visual acuity, IOP = intraocular pressure, pRPC = peripapillary radial peripapillary capillaries, pRNFL = peripapillary retinal nerve fiber layer, SQ = scan quality.

Vessel density and retinal layer thickness of the three groups are presented in Table 2. Statistically significant decreases in SVP vessel density and IRL thickness were found in the perifoveal and parafoveal areas in POAG compared to AD and HC, based on whole en face images (p<0.001 for all). Compared to POAG and HC, a significant decrease in vessel density was found in the DVP of the AD group as well as thinning of the ORL (p<0.05). The most noticeable DVP impairment in AD occurred in the perifoveal region compared to POAG and HC (p = 0.002 and p = 0.004, respectively), while in the ORL, the most noticeable reduction in thickness applies to the whole en face image (p<0.001 and p = 0.004, respectively).

**Table 2:** Vessel density and retinal layers thickness of the studied groups.

Parametr	Healthy	POAG	AD	P-Value† AD vs. POAG	P-Value† AD vs. Healthy	P-Value† POAG vs. Healthy
**SVP vessel density (%)**						
**whole**	48.5± 3.4	42.4±5.4	46.8±3.2	**<0.001**	0.766	**<0.001**
**fovea**	23.9±6.6	18.4±5.7	19.7±6.2	0.45	**0.011**	**<0.001**
**parafovea**	51.4±4.3	46.7±5.5	49.4±4	**<0.001**	0.68	**<0.001**
**perifovea**	48.8±3.6	42.7±5.9	46.8±3.3	**<0.001**	0.582	**<0.001**
**DVP vessel density (%)**						
**whole**	48.5±5.1	47.6±5.2	45±4.7	**0.014**	**0.032**	0.926
**fovea**	39.6±5.6	34.7±7.6	34.3±7.3	0.748	**0.005**	**0.006**
**parafovea**	53.2±3.4	53.5±4.1	51.7±3.6	**0.038**	**0.045**	0.21
**perifovea**	50±5.3	48.7±5.7	45.4±5.4	**0.002**	**0.004**	0.922
**IRL thickness (μm)**						
**whole**	100.3±8.6	81.3±12.1	93.5±7.7	**<0.001**	**0.032**	**<0.001**
**fovea**	60.8±9.4	49.7±9.2	53.5±7.9	0.077	**0.001**	**<0.001**
**parafovea**	110±9	89.9±14.8	102.5±9.8	**<0.001**	0.061	**<0.001**
**perifovea**	100.6±10.5	80.6±11.6	94.5±8.1	**<0.001**	0.066	**<0.001**
**ORL thickness (μm)**						
**whole**	202.4±7.2	207.4±8	195.7±7.5	**<0.001**	**0.004**	**0.01**
**fovea**	221.7±13.9	219.8±12.6	211.7±14.1	**0.023**	**0.005**	0.274
**parafovea**	215.9±8.1	217.8±8.7	208.9±8.8	**<0.001**	**0.007**	0.468
**perifovea**	184.2±6.9	184.5±6.8	178.6±6.5	**0.004**	**0.018**	0.748

Significant values appear in boldface.

Mean (standard deviation).

^†^P-values adjusted for age, inter-eye correlation and SQ (in OCTA), based on Linear Mixed Effects Model.

Abbreviations: POAG = primary open-angle glaucoma, AD = Alzheimer’s disease, SVP = superficial vascular plexus, DVP = deep vascular plexus, IRL = inner retinal layers, ORL = outer retinal layers, SQ = scan quality, OCTA = optical coherence tomography angiography.

The percentage loss of vessel density in the SVP and thickness of the IRL in AD and POAG are summarized in Table 3. Percentage losses of vessel density in SVP and IRL thickness were considerable in eyes with POAG compared to AD (p<0.001 for all, except SVP vessel density in the perifoveal area, where p = 0.003). The extent of IRL thickness percentage losses were significantly greater than corresponding percentage losses of vessel density in the SVP in the AD and POAG groups (p<0.001 for all, except the perifoveal region in AD, where p = 0.01).

**Table 3:** Percentage loss of vessel density in superficial vascular plexus and inner retinal layer thickness in the primary open-angle glaucoma and Alzheimer’s disease groups.

Parametr	Percentage loss†		P-Value
	SVP vessel density (%)	IRL thickness (%)	
**Whole image**			
**POAG**	11.1 (8.7–13.4)	17.22 (14.4–20.1)	**<0.001**
**AD**	0.9 (-0.7–2.5)	6.0 (3.7–8.3)	**<0.001**
**P-value**	**<0.001**	**<0.001**	
**Perifoveal**			
**POAG**	10.9 (8.3–13.6)	16.9 (14.0–19.7)	**<0.001**
**AD**	1.5 (-0.3–3.3)	4.6 (2.1–7.1)	**0.01**
**P-value**	**0.003**	**<0.001**	
**Parafoveal**			
**POAG**	7.6 (5.4–9.8)	17.2 (14.0–20.3)	**<0.001**
**AD**	1.19 (-0.8–3.2)	6.2 (3.6–8.7)	**<0.001**
**P-Value**	**<0.001**	**<0.001**	

Significant values appear in boldface.

^†^Percentage loss, which was calculated with the use of the Linear Mixed Effects Model, are shown in mean (95% confidence interval). The values of the percentage loss have been adjusted for the inter-eye correlation, age, gender and the scan quality (where applicable).

Abbreviations: POAG = primary open-angle glaucoma, AD = Alzheimer’s disease, SVP = superficial vascular plexus, IRL = inner retinal layers.

Table 4 summarizes the calculated percentage losses for vessel density in the DVP and thickness of the ORL in AD and POAG. Significantly greater losses of vascular density in the DVP and ORL thickness were observed in AD compared to POAG (p<0.001 for all, except DVP vessel density in the perifoveal area and whole en face images, where p<0.05). In addition, the extent of percentage losses in studied groups were similar (p>0.1, except whole en face images in POAG, where p = 0.005, and perifoveal area in AD, where p = 0.001).

**Table 4:** Percentage loss of vessel density in deep vascular plexus and outer retinal layer thickness in the primary open-angle glaucoma and Alzheimer’s disease groups.

Parametr	Percentage loss†		P-Value
	DVP vessel density (%)	ORL thickness (%)	
**Whole image**			
**POAG**	0.4 (-1.6–2.4)	-2.5 (-3.4- -1.6)	**0.005**
**AD**	4.9 (2.7–7.3)	3.2 (2.2–4.3)	0.158
**P-value**	**0.004**	**<0.001**	
**Perifoveal**			
**POAG**	0.8 (-1.3–2.9)	-0.7 (-1.6–0.2)	0.149
**AD**	6.9 (4.5–9.5)	2.4 (1.4–3.4)	**0.001**
**P-value**	**<0.001**	**<0.001**	
**Parafoveal**			
**POAG**	-1.3 (-2.97–0.3)	-0.7 (-1.7–0.2)	0.497
**AD**	1.8 (-0.1–3.6)	3.4 (2.2–4.6)	0.131
**P-value**	**0.015**	**<0.001**	

Significant values appear in boldface.

^†^Percentage loss, which was calculated with the use of the Linear Mixed Effects Model, are shown in mean (95% confidence interval). The values of the percentage loss have been adjusted for the inter-eye correlation, age, gender and the scan quality (where applicable).

Abbreviations: POAG = primary open-angle glaucoma, AD = Alzheimer’s disease, DVP = deep vascular plexus, ORL = outer retinal layers.

Table 5 shows associations between the presence of AD and POAG based on SD-OCT and OCTA measurements adjusted for age, sex, SQ, and inter-eye correlation using the GEE for multinomial responses. Positive associations among SD-OCT parameters were observed in the presence of AD, primarily loss of ORL thickness in each analyzed area where a 1% decrease of ORL thickness was associated with about 24–29% increase in odds of the presence of AD. According to OCTA measurements, a 1% decrease of vessel density in DVP was positively associated with a 4–9% increase in odds of the presence of AD. In POAG, the loss of pRNFL and IRL thickness measured by SD-OCT and the loss of vessel density in pRPC and SVP measured by OCTA measurements were positively associated with the presence of the disease. Conversely, in POAG, a 1% loss of pRNFL and IRL thickness measured by SD-OCT and a 1% loss of vessel density in the pRPC and SVP measured by OCTA were positively associated with a 13–23% increase in risk of presence of the disease.

**Table 5:** Multivariable analysis of the associations of 1% loss in vessel density and retinal layers thickness with the presence of Alzheimer’s disease and primary open-angle glaucoma.

Parameter	AD		POAG	
	OR per 1% loss	95% CI	OR per 1% loss	95% CI
**Optical coherence tomography angiography (vessel density)**				
**SVP whole**	1.03	(0.96–1.11)	**1.23**	**(1.13–1.33)**
**SVP perifoveal**	1.03	(0.98–1.09)	**1.17**	**(1.09–1.26)**
**SVP parafoveal**	1.03	(0.96–1.1)	**1.13**	**(1.06–1.2)**
**DVP whole**	**1.07**	**(1.02–1.13)**	1.01	(0.96–1.06)
**DVP perifoveal**	**1.09**	**(1.04–1.14)**	1.01	(0.97–1.06)
**DVP parafoveal**	1.04	(0.98–1.11)	0.97	(0.91–1.04)
**pRPC**	1.01	(0.93–1.09)	**1.23**	**(1.14–1.32)**
**Spectral-domain optical coherence tomography (thickness)**				
**IRL whole**	**1.09**	**(1.03–1.15)**	**1.21**	**(1.14–1.29)**
**IRL perifoveal**	1.04	(1–1.09)	**1.16**	**(1.1–1.23)**
**IRL parafoveal**	**1.09**	**(1.03–1.15)**	**1.19**	**(1.13–1.25)**
**ORL whole**	**1.29**	**(1.12–1.5)**	0.85	(0.74–0.97)
**ORL perifoveal**	**1.24**	**(1.07–1.43)**	0.97	(0.85–1.1)
**ORL parafovea**	**1.25**	**(1.09–1.44)**	0.97	(0.87–1.09)
**pRNFL**	1.04	(0.99–1.09)	**1.21**	**(1.14–1.28)**

Significant values appear in boldface.

Analysis adjusted for age, gender and scan quality.

Abbreviations: AD = Alzheimer’s disease, POAG = primary open-angle glaucoma, OR = odds ratio, CI = confidence interval, SVP = superficial vascular plexus, DVP = deep vascular plexus, IRL = inner retinal layers, ORL = outer retinal layers, pRPC = radial peripapillary capillaries, pRNFL = peripapillary retinal nerve fiber layer.

**Fig 2:**
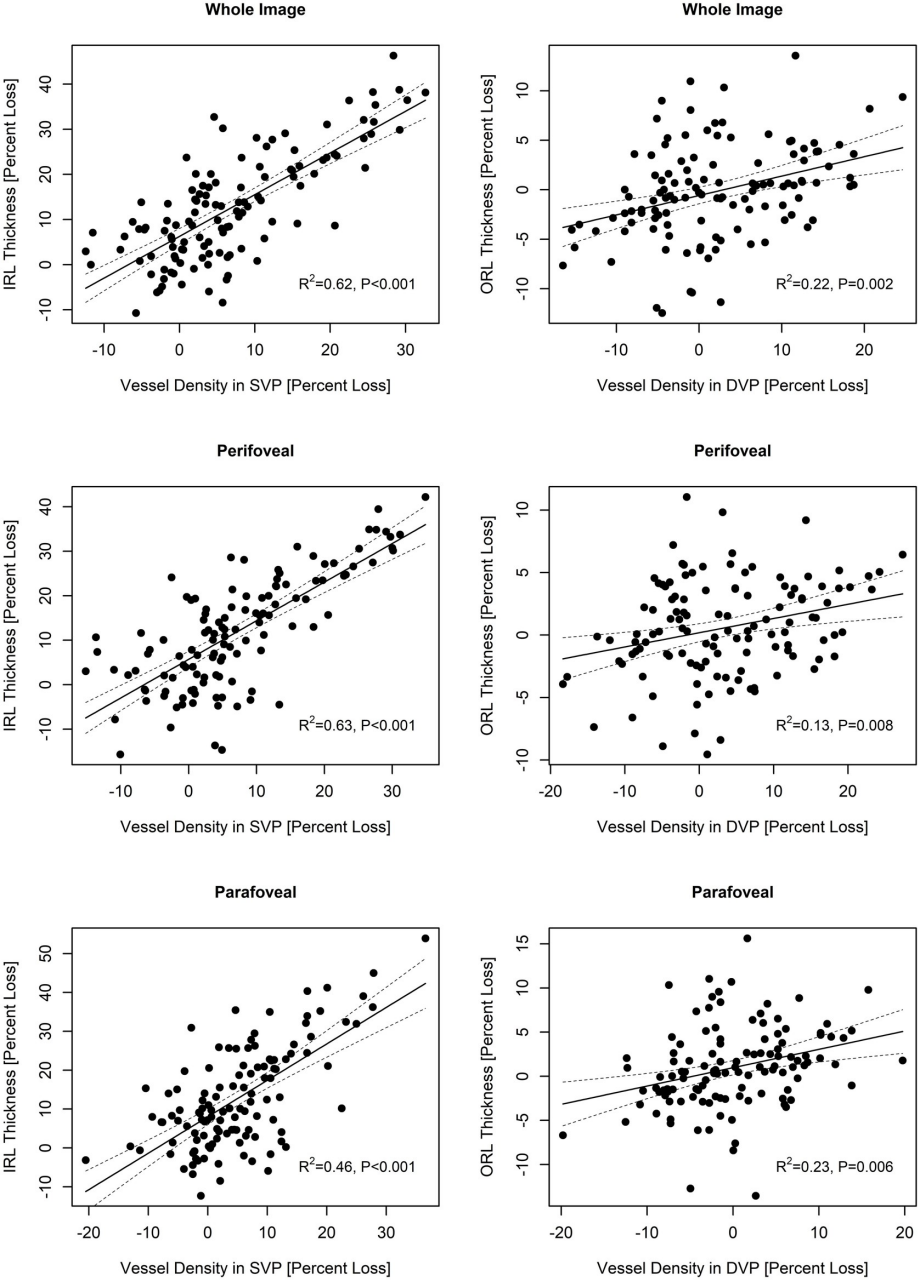
Scatterplots illustrating the correlation between percentage loss of retinal layers thickness and vessel density with linear regression curves in Alzheimer’s disease and primary open-angle glaucoma eyes.

Scatterplots presented in Fig 2 illustrate the association between percentage loss of IRL thickness and vessel density in the SVP and percentage loss of ORL thickness and vessel density in the DVP in AD and POAG. The observed correlation between percentage loss of IRL thickness and vessel density in the SVP (R2 values ranged from 0.43 to 0.63) was stronger than the association between ORL thickness and vessel density in the DVP (R2 values ranged from 0.13 to 0.23), however, all associations were statistically significant (Fig 2).

## Discussion

In this study, both ORL thickness and vessel density in DVP were significantly reduced in AD. A direct comparison of the percentage losses of vascular density and retinal thickness revealed a loss of ORL thickness and vessel density in DVP associated with the presence of AD, whereas a loss of IRL and pRNFL thickness and a loss of vessel density in SVP and RPC were associated with the presence of POAG. Analysis of the associations of 1% loss in vessel density and retinal layers thickness with the presence of AD shows positive associations primarily among SD-OCT parameters, where a 1% decrease of ORL thickness was associated with about 24–29% increase in odds of the presence of AD. We also confirmed that changes in retinal vasculature in SVP and DVP were respectively correlated with damage to the IRL and ORL in AD and POAG eyes.

Previous reports have indicated that RGC loss in AD may have a similar pathogenesis to POAG; therefore, the issue of common risk factors and mediators responsible for their emergence and development is increasingly raised [34]. Both diseases are characterized by initial changes in neuronal circuits and phosphorylation of mitogen-activated protein kinases. Propagation of neurodegenerative processes related to glial reaction, neuroinflammation, mitochondrial abnormalities associated with production of reactive oxygen species, and oxidative stress, etc., lead to apoptosis of nerve cells [35,36]. In addition, age is a common risk factor for neurodegenerative diseases and the RNFL is believed to naturally decrease with age at a rate of 0.44 μm per year [37]. Meta-analyses show that pRNFL thickness decreases in AD and MCI compared with HC [38,39]. Our results demonstrate a slight decrease in pRNFL thickness compared to HC, but much smaller than that in POAG eyes. Analysis of predictive factors for multinomial responses reveal no association between a decrease of pRNFL thickness and the presence of AD. Our findings are consistent with another large cohort study using advanced OCT that did not report association between dementia or MCI and pRNFL thickness [40].

The macula contains more than 50% of all RGCs, which have a cell body that is 10 to 20 times larger than the diameter of its axon [14]. In addition, structures of the GCL and IPL, containing RGC bodies and their dendrites, respectively, are characterized by less individual variability than the RNFL, which contains axons [41]. This suggests that measurements of retinal thickness in the macular region could be more useful than pRNFL thickness assessments for diagnosing and monitoring neurodegenerative diseases. In 2015, Cheung et al. used SD-OCT to show a decrease in GC-IPL thickness in the macula is more strongly associated with the presence of AD and MCI than a decrease of pRNFL thickness [14]. In other studies where the thickness of specific layers or full macular thickness was assessed, a relationship with the presence of AD was confirmed [40,42–44]. The results of our research reveal similar findings. We found a significant reduction of IRL thickness in whole en face images, but there was no difference in perifoveal and parafoveal areas compared to HC. Comparison of the percentage loss of IRL thickness between AD and POAG groups showed a greater percentage loss in POAG. We have also shown a significant relationship between the decrease of IRL thickness and the presence of AD, however, this association was lower than with the presence of POAG. A greater percentage loss of pRNFL and IRL thickness as well as a stronger relationship with the presence of the disease was observed in POAG compared to AD; therefore, we believe these parameters can be misleading when used to differentiate AD from POAG, and their use as a biomarker for AD is limited.

Previous studies have mainly focused on changes in IRL thickness, whereas few published reports have investigated the outer retinal metrics using SD-OCT. In AD, histological post-mortem studies of humans and animals models have revealed deposition of Aβ plaque in the posterior segment of the eye in various locations including the RNFL, GCL, IPL, OPL, and INL, in the photoreceptor outer segment layer of the retina, and some plaques were also observed in the sclera [45,46]. In addition, Aβ is deposited in the ORL as part of the aging process, where Aβ deposition has been noted in drusen, which can underlie the onset of age-related macular degeneration [47,48]. The extent to which ORL degeneration causes RGC neurotic changes remains unclear. A recent report that outer retinal degeneration may lead to dendritic RGC atrophy as a result of transneuronal changes in mice may explain some of the changes observed in our study [49,50]. We showed that patients with AD exhibit significant thinning of the ORL compared to eyes with POAG and HC, which is consistent with another study [51]. Our multivariable analysis of associations found that reduced ORL thickness is associated with a significant increase in the odds of the presence of AD. Comparison of percentage losses of IRL and ORL thickness demonstrated that the percentage loss of IRL is greater in AD eyes. However, direct comparison between the AD and POAG groups reveals that the percentage loss of IRL thickness is also greater in the POAG group, which suggests this parameter is associated more so with an increase in odds of the presence of POAG than AD. Uchida et al. quantitatively assessed changes in ORL using SD-OCT in various neurodegenerative diseases, including AD. In contrast to our study, they found no identifiable differences in ORL parameters across neurodegenerative disease groups and controls. This could be explained by several reasons: AD patients did not undergo PET imaging to confirm the presence of Aβ deposits; besides, SD-OCT examination was performed using a different device (Cirrus 4000 HD-OCT) and semi-automatic segmentation on the software platform was performed with manual correction to identify boundaries of interest; finally, thickness of the ORL (between the INL and RPE) was not assessed, but instead, thicknesses were measured from the ONL to ellipsoid zone and from the ellipsoid zone to RPE [52].

Post-mortem studies of patients with dementia have provided evidence that AD involves cerebrovascular pathology. Blood vessels of the retina and brain have common embryological origins and show anatomical and physiological similarities; therefore, retinal vascular examination may be valuable in providing new information on AD [17]. Bulut et al. were probably the first to use OCTA imaging to analyze vascular lesions of the retina in AD patients. They found a reduction in the density of vessels in SVP in the eyes of AD compared to HC [18]. Subsequent research groups confirmed a decrease in retinal vascular density in SVP in patients diagnosed with AD [53–55]. Jiang et al. found a slight decrease in GC-IPL thickness in AD compared with MCI and HC. In addition, they noted a reduction in vascular density in each retinal plexus in AD patients, with a significant correlation between vessel density in the DVP and retinal thickness of the GCL-IPL [56]. There are some doubts about the use of retinal vessel density as a specific biomarker for AD since earlier research on glaucoma confirmed the use of vascular density assessment in the diagnosis and monitoring of POAG. Studies using OCTA in POAG eyes have repeatedly provided evidence of microvascular dropout in the form of a decrease in vessel density within the ONH, the peripapillary retina, and the macula, primarily in the form of a decrease of vascular density in the SVP [20,57,58]. The present study quantitatively compared vascular parameters in the eyes of AD and POAG patients and confirmed previous reports that the density of vessels in the individual retinal plexuses are significantly different among AD and POAG groups. A significant reduction of vessel density in the DVP was observed in AD, whereas a significant decrease of vessel density in the SVP was noted in POAG. Since vessels of the SVP are located in the IRL between the ILM and outer boundary of the IPL, and vessels of the DVP are contained within the outer boundaries of the IPL and OPL, which belong to the ORL. We assessed the relationship between thickness of the retinal layers and density of vessels in their corresponding retinal plexuses and found a correlation between percentage loss of IRL thickness and vessel density in the SVP and between percentage loss of ORL thickness and vessel density in the DVP in both AD and POAG. We believe capillary impairment is associated with AD-mediated neurodegeneration, and it is possible that the retina is highly susceptible to DVP dysfunction in AD, which may indicate disease progression [59]. This is probably due to the diameter of the vessels: DVP vessels are thinner and have a smaller cross-section making them more sensitive to disease progression. Furthermore, Aβ plaques accumulating around the walls of vessels reduce the diameter of vessels leading to blood flow disorders, and also reduced angiogenesis, likely due to binding and blocking of vascular endothelial growth factor by Aβ deposits [18,60].

To compare different parameters with different units and potentially different dynamic ranges, we normalized measurements by calculating the percentage loss of deviation from the mean value of the HC group. By analyzing percentage losses, we were able to directly compare the thickness and density of vessels between groups. In this study, we demonstrated that in POAG eyes there are significant changes in the inner retina, and the percentage loss of IRL thickness was significantly greater than for SVP vessel density. It is different in the deeper layers of the retina, where significant changes are evident only in AD eyes. We found no differences in percentage loss, except in the perifoveal region, where we found a greater percentage loss of vessel density in DVP than of ORL thickness. Therefore, we believe the cause of neurodegeneration in AD may be different to that of POAG. Microvascular and thickness mismatch in POAG suggest that neurodegeneration may occur sooner and more quickly than vascular damage, which is consistent with another study; whereas, significant changes in eyes of AD patients primarily occur in deeper layers of the retina and the neurodegenerative changes may be secondary to microcirculation disorders where percentage loss of vessel density in DVP is greater than changes in ORL thickness in the perifoveal region [61].

The strength of our study is the fact that AD patients were accurately diagnosed through detailed neurocognitive testing and PET imaging with florbetapir F 18 radioligand analysis, which can readily differentiate participants with normal cognition from those with dementia due to AD. In addition, PET imaging enabled accurate differentiation of AD from dementias with different etiologies.

However, the present study had some limitations. It was a cross-sectional study precluding ability to study patients longitudinally, in addition the case-control design excludes full application in the real clinic population. Another limitation was the relatively small groups of subjects. Therefore, both eyes of some patients were included and the LMM was used which takes into account the correlation between repeated observations from the same individual (with application of the inter-eye correlation). We do not know the time of the disease evolution from its inception to the inclusion of the patient in the study, because most often at the beginning the course of these diseases is asymptomatic. Therefore, we included patients with POAG and AD in mild or moderate stage of disease. We excluded patients in severe stages of the disease that highlight the differences between the study groups. Despite the fact that screening of cognitive function with MMSE was performed for each patient along with a detailed fundus examination to rule out glaucomatous optic neuropathy, selection bias cannot be ruled out. Patients with AD did not undergo visual field testing owing to low reliability of the static perimetry test requiring concentration and cooperation of patients, and PET imaging was performed only in the group of AD patients because it is too expensive to be routinely used in screening tests [62,63]. Patients with POAG did not discontinue ocular hypotensive eye drops, which might affect ocular blood flow [64,65]. The effect of antihypertensive eye drops is likely to persist for 1–4 weeks from the time of withdrawal; therefore, for ethical and medical reasons, patients with POAG involved in the present study did not stop using them [66]. For the same reasons, the use of procognitive drugs in patients with AD has not been discontinued.

## Conclusions

New technologies such as SD-OCT and OCTA contribute to progress in the diagnosis of AD and a better understanding of its pathophysiology. Structural changes in the retina and its microcirculation may be directly related to the deposition of Aβ plaques. Unfortunately, structural changes found in the inner retina may be non-specific and are also common in glaucoma. Nevertheless, measurements of deeper retinal layers and analysis of vessel density in DVP could potentially improve diagnostic capabilities and may provide a valuable approach for predicting AD development. More research on a larger group of patients is required to make these methods more sensitive and specific enough to be useful in everyday practice.

## Supporting information

S1 Table Dataset. (XLSX)
